# Children's mobility and environmental exposures in urban landscapes: A cross-sectional study of 10–11 year old Scottish children

**DOI:** 10.1016/j.socscimed.2019.01.047

**Published:** 2019-03

**Authors:** Jonathan R. Olsen, Richard Mitchell, Paul McCrorie, Anne Ellaway

**Affiliations:** MRC/CSO Social and Public Health Sciences Unit, University of Glasgow, Glasgow, UK

**Keywords:** Children, Mobility, Epidemiology, Environment, Spatial epidemiology, Environmental exposure, Inequalities

## Abstract

Research into how the environment affects health and related behaviour is typically limited in at least two ways: it represents the environment to which people are exposed using fixed areal units, and, it focuses on one or two environmental characteristics only. This study developed a methodology for describing children's mobility and the complexity of their environmental exposure across a 1934 km^2^ study area, including urban, suburban and rural zones. It conceptualised and modelled this area as a landscape, comprised of spatially discrete amenities, infrastructure features, differing land covers/use and broader environmental contexts. The model used a 25 m^2^ grid system (∼3 million cells). For each cell, there was detailed built-environment information. We joined data for 100 10/11-year-old children who had worn GPS trackers to provide individual-level mobility information for one week during 2015/16 to our model. Using negative binomial regression, we explored which landscape features were associated with a child visiting that space and time spent there. We examined whether relationships between the features across our study area and children's use of the space differed by their sociodemographic characteristics.

We found that children often used specific amenities outside their home neighbourhood, even if they were also available close to home. They spent more time in cells containing roads/transportation stops, food/drink retail (Incidence rate ratio (IRR):4.02, 95%CI 2.33 to 6.94), places of worship (IRR:5.98, 95%CI 3.33 to 10.72) and libraries (IRR:7.40, 95%CI 2.13 to 25.68), independently of proximity to home.

This has importance for the optimal location of place-based health interventions. If we want to target children, we need to understand that using fixed neighbourhood boundaries may not be the best way to do it. The variations we found in time spent in certain areas by sex and socio-economic position also raise the possibility that interventions which ignore these differences may exacerbate inequalities.

## Highlights

• We describe children's mobility (using GPS) and their environmental exposures.
• We find that children won't necessarily use facilities within their home area.
• We developed a novel landscape model across a 1934km2 study area.
• The model highlights specific land uses where children spent more or less time.
• This is important for the optimal locating of place-based health interventions.

## 1 Introduction

Whilst the influence of environmental characteristics on health, over and above individual factors, is well established (Macintyre and Ellaway, 2003), precise understanding of how social and physical environments affect health remains elusive. In particular, clear and effective place-based interventions to maximise health and minimise health inequalities are rare. This is at least partly because of problems with how the field typically measures ‘exposure’ to environment and, at a deeper level, how it thinks about space and society.

Much research in this field has focused on an individual's residential ‘neighbourhood’ as the key areal unit of ‘exposure’. Neighbourhood is often defined and measured using static entities such as administrative units (e.g. census tracts, postal geography) (Van Ham et al., 2012), or pre-defined circular, network or polygon buffers placed around a home, which are assumed to represent their neighbourhood. Although such predefined units are useful to health researchers in that secondary sources on health and sociodemographic data are frequently available for them (Weiss et al., 2007), their weaknesses are obvious. Many people move around and are not confined to these static areal units; and just because people live within a particular areal unit does not mean they are exposed to all its environmental characteristics. Within health geography and epidemiology, for example, Chaix and colleagues have been particularly influential and focused on the challenges of measuring and modelling how individuals' day to day mobility affects the extent of their exposure to different environments and ability to lead a healthy life (Chaix et al., 2017; Kestens et al., 2017; Perchoux et al., 2013). Other influential researcher in terms of modelling mobility/exposure using areal units includes physical activity (Hillsdon et al., 2015), utilising GPS for health research (Jankowska et al., 2015; Krenn et al., 2011) and understanding the Uncertain Geographic Context Problem (UGCoP) (Kwan, 2012).

Whether, how and where people move within, and beyond, the area around their home will depend on both their individual characteristics, interests, abilities and affluence, but also on the wider environment itself. Literature on spatial mobility largely falls into two distinct categories: studies which describe *potential path areas*, that is the area and locations which individuals *could* have visited subject to time and other (e.g. transportation availability) constraints (Patterson and Farber, 2015); and studies which describe people's *activity spaces*, that is all the locations which individuals *actually* went to. Potential path areas are sometimes based on pre-defined home or work network buffers (Karusisi et al., 2013; Prins et al., 2012; Roemmich et al., 2006). At their most basic, these are really just different ways to create static neighbourhood boundaries; they still result in a fixed areal unit. The availability of precise location technologies, such as Global Positioning System (GPS) devices has increased (McCrorie et al., 2014), and the number of studies using them to describe activity space is growing. Epidemiology has used these tools to explore environmental exposures. It is argued that measurement of visits or proximity to destinations recorded via GPS devices provides more accurate measure of exposure (Burgoine et al., 2015; Hillsdon et al., 2015; Klinker et al., 2014). This is as much because the data from GPS devices tell us how long someone was in a particular location, as it is because of locational accuracy. Time *and* space are both significant components of exposure to risk or protective environments.

Although studies using GPS highlight that pre-defined neighbourhood area units may be inappropriate for many (though not all), as some groups of people are highly mobile across urban areas (Patterson and Farber, 2015; Yin et al., 2013), this analytical approach still dominates empirical studies. Recent review articles argue against it (Patterson and Farber, 2015). The potential for misrepresentation of how people and space interact matters because it both hampers our understanding of the relationships between people and their environments, and because misunderstanding of exposure by researchers could lead to ineffective policymaking (Sadler and Gilliland, 2015). Studies found that for adolescents in the United States, and children living in New Zealand and Canada, 18% of total moderate-to-vigorous physical activity, 38% of leisure time and 24% of out-of-school-time was not within or near to the home or school ‘neighbourhood’ (Carlson et al., 2015; Chambers et al., 2017; Loebach and Gilliland, 2016). It is clear that the ‘traditional’ assessment of children's mobility and environmental exposure using fixed neighbourhood boundaries is not effective.

A second major challenge in researching how environment affects health is dealing with the fact that our environments are incredibly complex and multifaceted. Whilst epidemiology has developed increasingly effective methods for isolating causal relationships between single environmental attributes and health outcomes, the utility of these approaches for helping solve the most pressing ‘wicked’ social and health problems is questionable (Krieger and Davey Smith, 2016). No one is only exposed to single environmental attributes and the relationships between environmental characteristics and human health and behaviour are dynamic and complex. Although our analyses do not explicitly contribute to the understanding human mobility within the field of complexity science, we do wish to draw on its overarching theory to try to advance thinking and approaches to assessing human/environment interactions.

We have taken inspiration from ecology, which has developed both methods and theory for considering how animals move and interact within ecosystems (Turner et al., 2001). Ecology, has led the development of methods to capture and analyse animal positions, providing a unifying paradigm for movement paths to show how and where individuals interact with the *whole* ecosystem around them (Cagnacci et al., 2010). Further, landscape ecology recognises that organisms and their habitats exist in a complex (eco)system in which the presence, size, shape, spatial arrangement of, and balance between, different kinds of habitats affect organisms' health-related outcomes (Gergel and Turner, 2017). If we conceptualise a landscape as a complex mix of discrete and continuous environmental components and resources, it is not too great a leap to see how this might apply to human settlements too and this has previously been referred to as human ecology (Lawrence, 2018). We can conceptualise villages, towns and cities as a single ‘landscape’ rather than as an assembly of fixed and non-fluid neighbourhoods. Thinking of the city as a spatially heterogeneous mosaic of interacting ‘habitats’ in and through which residents live and move allows that, while some people have a very tightly defined area around their home as their neighbourhood, for many others their neighbourhood encompasses a large swath of the urban area. Just as in the natural world, a human landscape contains different kinds of habitats; not all ‘species’ use/pass through all parts of the landscape (Fahrig et al., 2011). Conceptualising space in this way focuses attention on *all* habitats, all parts of the urban area and all aspects of the built environment that constitute the urban fabric (Ersoy et al., 2015). When we add information on individual's time/space geography to this ‘landscape’, in effect we are able to create ‘personalised activity spaces’ which assess all the types of environments people visit. In turn, when we have these activity spaces for large numbers of people, in different urban environments, we can compare how the use of landscape varies by characteristics of individuals (e.g. age, sex, socio-economic position, and residing in more affluent or deprived areas), and also assess how urban form itself might affect where people go, and what they are able to do.

This approach was inspired by, and based on, Species Distribution Models (SDMs) frequently used in ecological research (Guisan and Thuiller, 2005). SDMs are created by building a ‘raster stack’; overlaying multiple detailed geographic data sets, summarising them at grid cell scale and then overlaying detailed mobility information. SDMs combine concepts from ecology, statistics and information technology to provide responses of species to their environments based at an individual level rather than community (Elith and Leathwick, 2009). Although SDMs are frequently used, the size of the grid cell is subject to the species under investigation and requires sensitivity analysis.

### 1.1 Study aims and objectives

The aims of this study were:

(a) Create a fine scaled digital model of a complex urban landscape in terms of its land-use and the availability of facilities and amenities;
(b) Using GPS data, describe which features of the urban landscape were associated with children visiting particular locations within it, and with time spent there,
(c) Explore variation in these relationships by individual characteristics (sex and socio-economic status) and area deprivation, and;
(d) Assess access and use of facilities within the home neighbourhood and wider urban landscape.

## 2 Materials and methods

### 2.1 Study participants

We analysed children from the ‘Studying Physical Activity in Children's Environments across Scotland’ (SPACES) study (http://spaces.sphsu.mrc.ac.uk/). The children involved in SPACES were recruited from the Growing up in Scotland (GUS) study, a nationally representative longitudinal cohort study originating in 2005. As part of the annual data collection (conducted between September 2014 and February 2015 when the children were aged approximately 10 years old), parents and children were provided with brief information about the SPACES study and asked if their contact details could be passed on to SPACES staff. From a possible 2402 children who had participated in GUS age 10 (year) interviews, 90% (n:2162) of parents consented to be contacted by the SPACES team, and were sent study information, registration documents, and consent forms by post using the main parent/carer as primary contact.

#### 2.1.1 Ethical approval

The data collection for SPACES took place between May 2015 and May 2016 and ethical approval was provided by the College of Social Sciences, University of Glasgow (CSS ref: 400140067).

### 2.2 Location measurement using Global Positioning System (GPS) device

Children who consented to participate in the study were provided with a GPS device (Qstarz BT-Q1000XT; Qstarz International Co., Ltd, Taiwan) and asked to wear the device over eight consecutive days during the waking hours. The GPS devices have a median location error of 2.5 m and are found to be acceptable for use in larger population studies, especially with relatively long data collection periods (7 days or more) (Schipperijn et al., 2014). The device recorded the child's location every 10 s. We refer here to each location recorded as a ‘point’.

### 2.3 Additional variables

The child's home and school location were collected, as well as age at data collection and information describing sex and parental education attainment level (no qualification, lower level standard grades, upper level standard grades, higher grades or degree level). The area-based relative deprivation status of the child's home location was also attached using the 2012 Scottish Index of Multiple Deprivation (SIMD) (Scottish Government, 2012). The SIMD combines 38 indicators across the 7 domains (income, employment, health, education, skills and training, housing, geographic access and crime), and then categorises multiple deprivation scores into quintiles using a ranking approach (1 = most deprived, 5 = least deprived).

### 2.4 Design and study area

#### 2.4.1 Creating complex urban landscape

We used Geographical Information Systems (GIS; ArcMap 10.3 [ESRI, California]) to construct a model of the urban landscape within the Central Belt of Scotland. The model comprised a 25 m^2^ grid system (we also undertook sensitivity analysis of grid size, described later). For each cell, we captured detailed land-use information such as the presence of: roads, retail outlets, leisure centres, and greenspace, together with other contextual information such as walkability measures and socio-economic deprivation (full description, Table 1). This created a comprehensive land-use description for each small piece of the landscape mosaic.

**Table 1:** Land-use and contextual variables captured for every grid cell.

Variable	Type	Classification	Source
**Land-use variables**			
Motorway or A road	Line	Binary	Ordnance Survey Integrated Transport Network
B or minor road	Line	Binary	Ordnance Survey Integrated Transport Network
Railway stop	Polygon	Binary	Ordnance Survey Open Map - Local
Bus stop	Point	Binary	UK Department for Transport, National Public Transport Access Nodes
Food and/or drink retail	Point	Binary	Ordnance Survey Points of Interest (Classification: Food, Drink or Multi item retail)
Primary School	Polygon	Binary	Ordnance Survey Open Map - Local
Leisure Centre	Polygon	Binary	Ordnance Survey Open Map - Local
Place of worship	Polygon	Binary	Ordnance Survey Open Map - Local
Library	Polygon	Binary	Ordnance Survey Open Map - Local
Derelict land	Polygon	Binary	Scottish Greenspace Map
Private Gardens	Polygon	Binary	Scottish Greenspace Map
Playing field	Polygon	Binary	Scottish Greenspace Map
Sports club	Polygon	Binary	Scottish Greenspace Map
Woodland	Polygon	Binary	Scottish Greenspace Map
Public park	Polygon	Binary	Scottish Greenspace Map
Play park	Polygon	Binary	Scottish Greenspace Map
Green verge	Polygon	Binary	Scottish Greenspace Map
Other	Polygon	Binary	Scottish Greenspace Map
**Contextual variables**			
Dense population	Area based (Scottish datazone)*	Binary	Scottish Government 2013 Datazone population (>= 2677 per km^2^)
Urban	Area based (Scottish datazone)*	Binary	Scottish Government 6 fold Urban/Rural classification (Classified as Urban 1 or 2)
Income SIMD	Area based (Scottish datazone)*	Quintile: 1 = most deprived, 5 = least deprived	Scottish Government SIMD 2012
Walkability score (defined using a composite ‘walkability score’ based on street/path connectivity, and dwelling density)	Area based (Scottish datazone)*	Quintile: 1 = most walkable, 5 = least walkable	Macdonald et al. (2016)

Note: *Binary outcome* 1/0 indicates yes/no to presence of variable. *Type* defines geographical shape file type. *Based on datazone area classification centroid of grid cell was within.

**Fig. 1:**
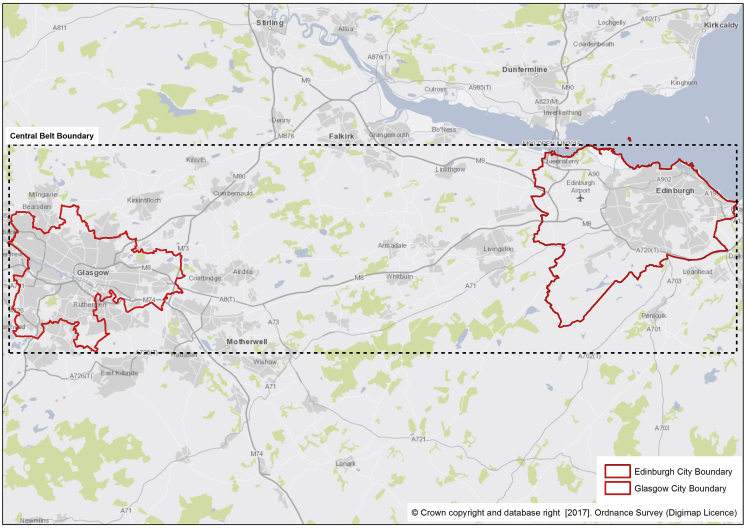
Scottish Central Belt Boundary.

The grid system was constructed for the Central Belt of Scotland, an area incorporating the administrative boundaries of Glasgow and Edinburgh Cities (Fig. 1). By including two major cities and the urban/rural hinterlands between, we created a varied geographical landscape to develop our methods and ideas. The cities of Glasgow and Edinburgh both contain areas which are amongst the most and least deprived areas in Scotland (The Scottish Government, 2016). In total the central belt contained 3,202,940 cells. SPACES children were included in the analysis if their home location was within the Central Belt boundary (n:100), 96.4% of all GPS points for these children were within this boundary.

##### 2.4.1.1 Geoprocessing of GPS data

Qstarz devices record their positional accuracy via positional dilution of precision (PDOP) values (Langley, 1999); a measure that uses the number and alignment of available satellites to determine the expected uncertainty of a GPS data point. Following established protocols (Schipperijn et al., 2014), PDOP values of <10 were identified as valid and used for further analyses. Each child's GPS data were converted from a raw text file to a shapefile and then projected to British National Grid coordinate system using R 3.4.1 (R Institute, Vienna, ggmap). Once projected, individual GPS data were spatially joined to a Central Belt grid (every child had their own grid model) and a count of their GPS points within each cell was calculated. Given that each point represented the child's location for a 10 s time period, this count could be converted to ‘time spent there’ for each cell.

We identified the grid cells that were within 50 m of the child's home postcode, and those which intersected the child's school location (using a polygon of the schools geographical boundary (source: Table 1)). We also calculated the linear distance (meters) of *all* grid cells (using a cell centroid) to the child's home location using the ‘*near proximity tool*’ within ArcMap 10.3.

##### 2.4.1.2 Land-use and contextual definitions

Each grid cell was populated with land-use and contextual data based on its spatial location (these attributes are listed in Table 1). In most cases these attributes were available as vector map files and we joined them to the grid cells using the intersect tool in ArcMap 10.3. Each land-use was spatially joined separately as an individual column within the dataset. If, for example, the grid cell contained a motorway, that was recorded as a ‘yes’ value in the ‘motorway’ column of the grid's attribute table. Cells were thus able to have multiple characteristics and thus reflect the complexity of the urban landscape. A cell might, for example, be identified as containing a road, a bus stop, and a green verge.

##### 2.4.1.3 Sensitivity analysis

Getting the grid cell size correct was important. If the cell size was too large it could contain too great a number of land-uses, making interpretation of results difficult. For example, if the cell contained both a park and a leisure centre we would be unable to tell which of these the child had been in the cell to visit. In contrast, very small grid cells would create an enormous dataset and create processing problems.

**Fig. 2:**
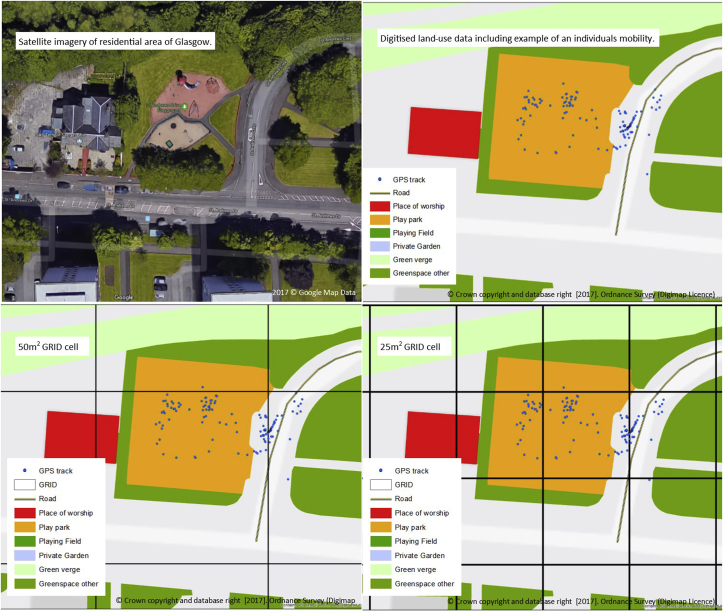
Grid cell boundaries, land-uses and GPS tracks.

We tested two grid cell sizes, 25 m^2^ and 50 m^2^, to compare their ability to capture land-use and infer the reason/activity a child was in that location for. The 25 m^2^ grid cell size was chosen based on the accuracy of the GPS devices, 78.7% of GPS points are expected to fall within 10 m of the precise location (Schipperijn et al., 2014). We also chose to compare with a grid cell size double this to assess whether a larger boundary would be accurate for inferring the place of the child's location and provide a lower number of grid cells to improve statistical processing time. Fig. 2 shows four images of the same geographical area: (a) an aerial image of the area, (b) a digitised map highlighting the land uses captured by our model in this area, and an example of a child's GPS points on the playing field, (c) the information in map b with a 50 m^2^ grid overlaid, and (d) the information in map b with a 25 m^2^ grid overlaid. Following detailed visual inspection of a subsample of study participants' (n = 30) GPS points at various amenities, we chose to adopt the 25 m^2^ grid size as this seemed to provide a better discrimination between the land-uses that might be matched to the GPS points. This discrimination is illustrated in Fig. 2 (c) and (d), which shows the smaller 25 m^2^ grid cell discriminates better between the land-uses associated with the GPS points. It's clear from the GPS points that time was spent in the playing field, rather than the place of worship; the 25 m^2^ cells work better to distinguish this, whereas the 50 m^2^ cell includes both land-uses. Our sensitivity analyses suggested 25 m^2^ was a good compromise between efficiency and accuracy.

### 2.5 Statistical analysis

#### 2.5.1 Descriptive analysis

We calculated the amount of time children spent at their home, school and outside of these two locations. We also described the number of land-use characteristics by grid cells across the central belt area of Scotland, and Glasgow and Edinburgh cities only, excluding underlying contextual attributes such as walkability score and income SIMD. We described the total number of built environment attributes within grid cells that children spend time in, examining only grid cells outside of the home and school location, as these environments are not visited by ‘choice’.

#### 2.5.2 Main effect models

Our main study outcome was count of GPS points within a cell, representing time spent there. We aimed to assess relationships between time spent and what the cell contained. As our outcome measure represented counts, and overdispersion within the dataset, we used a negative binomial regression model rather than poisson. A zero-inflated negative binominal regression model was considered but not chosen. Distance from the children's homes was a strong driver of zero counts (70% of all GPS points were within either the home or school boundary (Table 2)). We therefore controlled for distance from home with the negative binomial models and structured the analysis to perform these models both unadjusted and adjusted for distance from home. The datasets for each child were merged into a single dataset for analysis, and models were subsequently adjusted for clustering by individual in the models. Total count of points within a cell was the dependent variable, with the contextual and land-uses (Table 1) the independent variables. Since all contextual and land-use characteristics were in the model, the influence of one characteristic on time spent was, in effect, adjusted for all others.

**Table 2:** Sociodemographic characteristics and summary of GPS data of study participants.

Variable	Central Belt (excluding Glasgow and Edinburgh)	Edinburgh	Glasgow	All
**Sex**				
Male	27	12	11	50
Female	29	16	5	50
**Age (years)**				
10	34	13	9	56
11	22	15	7	44
**BMI classification**				
Underweight	1	–	–	1
Healthy weight	37	17	13	67
Overweight	7	6	2	15
Obese	11	5	1	17
**Household Socioeconomic status quintile**				
1 (Most Deprived)	4	3	2	9
2	10	–	5	15
3	11	–	–	11
4	12	2	2	16
5 (Least Deprived)	19	23	7	49
**Highest household qualification**				
Degree level	29	20	11	60
Higher grade (English A-level equivalent)	12	7	3	22
Upper level standard grade (English GCSEs at grade A* – C)	8	–	1	9
Lower level standard grade (English GCSEs at grade D – G)	5	–	1	6
No qualification	1	–	–	1
Other	1	–	–	1
Missing (data not provided by participant)	–	1	–	1
**GPS points**				
**Total gps points**	**1398617**	**1050134**	**305217**	**2753968**
*as hours*	*3885.0*	*2917.0*	*847.8*	*7649.9*
**GPS points at home**	**582639**	**586157**	**101367**	**1270163**
% of total points	41.7%	55.8%	33.2%	46.1%
**GPS points at school**	**376574**	**201754**	**80976**	**659304**
% of total points	26.9%	19.2%	26.5%	23.9%
**GPS points outside of school and home**	**439404**	**262223**	**122874**	**824501**
% of total points	31.4%	25.0%	40.3%	29.9%

We performed models in three stages to examine the influence of the home and school location, and distance from home on where children are likely to spend time: (1) without adjustment for proximity of a cell to school or home, (2) adjusting for proximity of a cell to home or school, and (3) adjusting for proximity of a cell to home or school, and distance of each cell from home. All models were performed unadjusted and adjusted for season in which the child wore the GPS device, and the child's sex, age, and socio-economic position (parent education and area-based SIMD).

#### 2.5.3 Interaction models

Previous research has shown that the amount of time children spend in places relating to physical activity varies by sex and socio-economic position (Jones et al., 2009; Wheeler et al., 2010). Therefore, we assessed whether the association between particular land-use attributes and time spent varied by sex and by socio-economic position. These attributes were leisure centres, private gardens, playing fields, public parks and play parks. We used the full model, adjusting for individual characteristics, home location, school location and distance from home and assessed interaction using a Wald test. Sparse data compelled us to model socio-economic position as a binary measure contrasting ‘deprived’ (those residing within the first or second most deprived quintiles) with ‘not deprived’ (quintiles three to five). All analyses were undertaken using Stata/SE 14.2 (StataCorp., College Station, Texas).

## 3 Results

### 3.1 Urban landscape characteristics

**Fig. 3:**
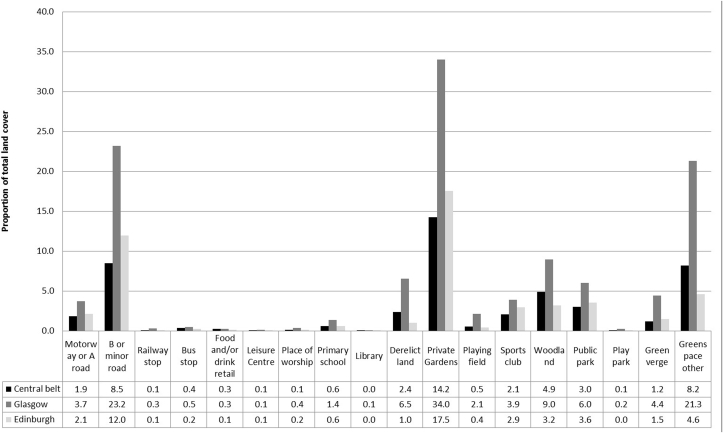
Proportion of landscapes containing specific land-use characteristics across Central Belt, Glasgow and Edinburgh City.

Fig. 3 displays the land-use characteristics as a proportion of total land-cover for the whole Central Belt of Scotland (including Glasgow and Edinburgh), and Glasgow and Edinburgh cities individually. For each of these three areas there were differences in the availability of specific land-use types. For example, Glasgow had a greater proportion of grid cells containing private gardens than Edinburgh.

**Fig. 4:**
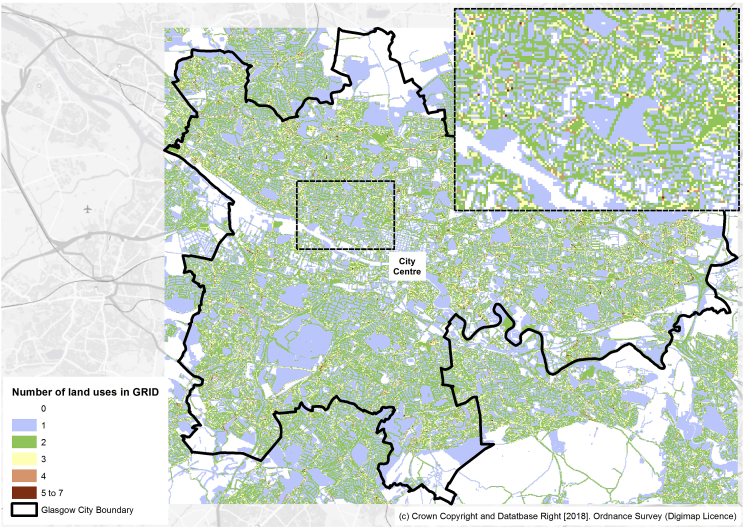
Geographical variation of number of land-uses by grid cell in Glasgow. Note: Excludes contextual data layers in grid cell count.

Grid cells, particularly when located in urban centres, often contained multiple land-uses: Fig. 4 provides a detailed map of Glasgow City in which each grid cell is categorised according to the number of land-uses it contained (detailed map of Edinburgh provided in Supplementary Materials). Residential areas of Glasgow, surrounding the city centre, have a greater number of land-uses within a grid cell compared to other non-residential areas of the city.

Examining only grid cells outside of the home and school location, there were a total of 824,501 GPS points. 41.7% (n:344,226 of GPS points outside of home/school) of these were in grid cells containing only one land-use and 43.7% (n:360,113 of total GPS points outside of home/school) were in grid cells containing 2 or more land-uses (maximum 6).

### 3.2 SPACES participants

In total, 100 children lived within the Central Belt grid and were subsequently included in the analysis. The sample contained an equal number of boys and girls, and a slightly higher proportion were aged 10 (56%, n:56) than 11, at the date they wore the GPS device (Table 2). The children represented a relatively affluent sample of the population; most children lived in the least deprived areas of Scotland (49%, n:49) and at least one member of the household was educated to degree level (60%, n:60). A total of 2,753,968 GPS points were recorded, 46.1% and 23.9% of points were within grid cells containing the home or school location, 29.9% outside of these two locations. A total of 7649.9 h of location recording was available for the 100 children.

### 3.3 Children's land-use exposures across urban landscapes

Table 3 presents the results of the negative binomial regression analysis of GPS counts within grid cells and the land-uses the cells contained, adjusted for individual characteristics (sex, season, parent education) and area deprivation of the home location (the unadjusted models are presented in Supplementary Table 1). In the adjusted analysis, the model coefficients were exponentiated to present incidence rate ratios (IRR) that represent the effect of a grid cell containing that land-use on the child spending time there (i.e. a greater count of GPS points), adjusted for all other land-uses we described in the urban landscape, the child's home location, school location and distance from home.

**Table 3:** Land-uses within grid cells associated with children spending time there (adjusted for sex, season, home SIMD, parent education).

Variable	Unadjusted				Adjusted for home & school				Adjusted for home, school and distance from home			
	IRR	P	LL 95% CI	UL 95% CI	IRR	P	LL 95% CI	UL 95% CI	IRR	P	LL 95% CI	UL 95% CI
Dense population	1.19	0.45	0.76	1.84	0.94	0.77	0.62	1.42	1.16	0.55	0.70	1.93

Urban (6 fold 1&2)	7.45	0.00	4.23	13.11	5.86	0.00	3.70	9.30	3.05	0.00	1.94	4.78

Income SIMD												
1 (Most deprived)	REF				REF				REF			
2	1.52	0.16	0.85	2.71	1.17	0.55	0.70	1.95	1.18	0.51	0.72	1.93
3	2.68	0.00	1.40	5.11	2.15	0.01	1.22	3.79	2.32	0.01	1.23	4.37
4	4.68	0.00	2.46	8.92	3.93	0.00	2.08	7.43	1.93	0.01	1.15	3.25
5 (Least deprived)	3.60	0.00	1.84	7.05	3.84	0.00	2.06	7.14	2.43	0.00	1.36	4.36

B or minor road	2.40	0.00	1.65	3.48	2.62	0.00	2.02	3.40	1.83	0.00	1.35	2.47

Motorway or A road	5.35	0.00	3.16	9.04	7.89	0.00	5.32	11.68	25.93	0.00	7.40	90.87

Railway stop	1.95	0.04	1.03	3.68	4.60	0.00	2.30	9.20	3.67	0.00	1.62	8.31

Bus stop	1.21	0.31	0.84	1.74	3.00	0.00	2.08	4.32	1.89	0.00	1.45	2.47

Walkability score												
1 (Least walkable)	REF				REF				REF			
2	9.22	0.00	5.86	14.52	6.30	0.00	4.01	9.84	4.60	0.00	2.99	7.10
3	12.96	0.00	8.06	20.82	10.25	0.00	6.62	15.77	9.20	0.00	4.84	17.49
4	19.63	0.00	10.43	36.94	9.41	0.00	5.58	15.83	6.00	0.00	3.16	11.38
5 (Most walkable)	22.07	0.00	10.45	46.61	19.20	0.00	10.21	36.09	9.73	0.00	4.79	19.80

Food and/or drink retail	2.37	0.04	1.04	5.41	5.15	0.00	2.86	9.24	4.02	0.00	2.33	6.94

Leisure Centre	5.65	0.00	2.66	12.00	8.81	0.00	2.89	15.55	14.86	0.15	0.37	590.62

Place of worship	6.34	0.00	3.06	13.16	14.16	0.00	6.96	28.76	5.98	0.00	3.33	10.72

Library	33.27	0.00	8.22	134.60	22.89	0.00	8.64	60.58	7.40	0.00	2.13	25.68

Derelict land	0.28	0.00	0.12	0.67	0.39	0.00	0.21	0.74	0.48	0.01	0.27	0.85

Private Gardens	4.45	0.00	3.05	6.48	1.40	0.03	1.04	1.88	0.83	0.16	0.63	1.08
Playing field	2.98	0.00	1.78	4.98	4.85	0.00	2.91	8.08	231.77	0.14	0.16	330489.44
Sports club	1.09	0.86	0.43	2.77	2.23	0.05	0.99	5.00	1.40	0.31	0.73	2.68
Woodland	0.28	0.00	0.19	0.40	0.46	0.00	0.33	0.64	0.41	0.00	0.31	0.53
Public park	4.51	0.00	2.23	9.13	6.14	0.00	3.26	11.53	5.63	0.07	0.88	36.22
Play park	1.25	0.58	0.56	2.79	4.86	0.01	1.37	17.18	2.25	0.20	0.66	7.73
Green verge	0.36	0.01	0.17	0.73	0.51	0.00	0.33	0.80	0.69	0.18	0.41	1.18
Other	0.98	0.90	0.66	1.43	1.43	0.06	0.99	2.08	1.31	0.15	0.91	1.88

School (polygon)					78433.00	0.00	43088.01	142914.24	11068.68	0.00	5276.17	23220.71

Home (50 m of postcode)					192914.04	0.00	136899.18	274306.12	42090.19	0.00	24684.97	71768.43

Distance from home (m)									1.00	0.00	1.00	1.00

Notes: IRR, Incidence Rate Ratio; LL 95% CI, Lower Level 95% Confidence Interval; and UL 95% CI, Upper Level 95% Confidence Interval.

The results highlight that ‘home’ (IRR:42090.19, 95% CI 24684.97 to 71768.43) and ‘school’ (IRR:11068.68, 95% CI 5276.17 to 23220.71) are, unsurprisingly the strongest predictors of where children spend time. Time spent in a cell fell as its distance from home increased. However, specific land-uses within a grid cell remained associated with children spending more time there, regardless of distance from home.

#### 3.3.1 Transport

There was a relationship between increased walkability of an area and a greater time spent in cells there, compared to the least walkable areas. Children also spent greater time in cells which contained roads, which may reflect a greater time overall spent within urban areas. When adjusted for distance from home, a cell containing an A-road or Motorway was associated with a greater amount of time spent (IRR:25.93, 95% CI 7.40 to 90.87), which may indicate that when outside of the immediate home area children spend a substantial amount of time travelling to other destinations. Once adjusted for home location, school location and distance from home, children also spent more time in cells containing rail (IRR:3.67, 95% CI 1.62 to 8.31) and bus stops (IRR:1.89, 95% CI 1.45 to 2.47).

#### 3.3.2 Facilities and other land characteristics

The presence of a library (IRR:7.40, 95% CI 2.13 to 25.68) and place of worship (IRR:6.32, 95% CI 2.63 to 11.88) within a grid cell were associated with more time spent there. Once adjusting for home and school location, children also spent more time in cells containing food and/or drink retail outlets (IRR:4.02, 95% CI 2.33 to 6.94) and this relationship remained after adjustment for distance from home. The presence of derelict land in a cell was not related to time spent there (IRR:0.48, 95% CI 0.27 to 0.85).

#### 3.3.3 Green space

Having playing fields and public parks, or play parks in a cell was associated with children spending time there in the unadjusted, and adjusted for home and school models respectively. However, once adjusting for distance from home these relationships were no longer significant.

### 3.4 Differences in land-use exposure by sex and socio-economic status

For most land-uses there were no significant interactions between time spent and sex, except for leisure centres where girls spent 52 times additional time at these places than boys (IRR:52.86, 95% CI 2.64 to 1058.56) (Table 4), albeit displaying wide confidence intervals. Children spent more time in cells in more affluent areas (regardless of the SIMD status of their own home location) (Table 3). The relationship between presence of playing fields in a cell, and time spent there was much stronger for children living in deprived areas than those from less deprived areas (IRR:1274.11, 95% CI 14.59 to 111301.72), as was the relationship between presence of a play park in a cell, and time spent there (IRR:62.80, 95% CI 14.44 to 273.14), again each interaction displaying wide confidence intervals.

**Table 4:** Interactions between sex, socio-economic status and visits to land-uses.

	Adjusted for home and distance from home			
	IRR	P	LL 95% CI	UL 95% CI
**i) Interactions by sex**				
**Leisure centre**				
Male	REF			
Female	52.86	0.01	2.64	1058.56
**Private gardens**				
Male	REF			
Female	1.05	0.88	0.57	1.93
**Playing fields**				
Male	REF			
Female	0.01	0.24	0.00	29.20
**Public Park**				
Male	REF			
Female	0.19	0.50	0.00	22.51
**Play park**				
Male	REF			
Female	1.02	0.99	0.10	9.82
** **	**Adjusted for home and distance from home1**			
	**IRR**	**P**	**LL 95% CI**	**UL 95% CI**
**ii) Interactions by socio-economic status**				
**Leisure centre**				
Less deprived	REF			
Most deprived	0.46	0.76	0.00	62.80
**Private gardens**				
Less deprived	REF			
Most deprived	1.38	0.30	0.75	2.51
**Playing fields**				
Less deprived	REF			
Most deprived	1274.11	0.00	14.59	111301.72
**Public Park**				
Less deprived	REF			
Most deprived	0.28	0.32	0.02	3.42
**Play park**				
Less deprived	REF			
Most deprived	62.80	0.00	14.44	273.14

Note: Interaction models included adjustment for all land-use characteristics in main outcome model.

### 3.5 Assessment of access to, and use of, facilities by children within a defined home neighbourhood and wider urban area

Table 5 highlights the availability and use of four key local facilities within a traditionally fixed definition of ‘neighbourhood’ - an 800 m centric buffer around the home (800 m buffer is a commonly used measure and approximates a 10 min walk (Harrison et al., 2011)). Of the 100 children, 32% (n = 32) had a leisure centre within their ‘neighbourhood’; however only 18.8% of them (n:6/32) actually visited this facility (as identified using GPS tracks). From the same 32 children, 31.3% (n:10/32) visited a leisure centre outside of their ‘neighbourhood’. A similar pattern is evident when exploring availability and visits to playing fields, public parks and libraries (Table 5).

**Table 5:** Accessibility to facilities within 800 m of home, visit to facility (GPS recorded) and visit to facility outside of 800 m home buffer.

Facility	Access to facility within 800 m home (n = 100)	If child had access, did they visit the facility?	If child had access, did they visit a facility outside of 800 m
Leisure Centre	32	6 (18.8%)	10 (31.3%)
Playing fields	44	16 (36.4%)	20 (45.5%)
Public park	83	49 (59.0%)	50 (60.2%)
Library	15	12 (29.3%)	10 (24.4%)

## 4 Discussion

In the introduction, we highlighted that traditional exposure assessments such as buffers around the home or fixed areal units are problematic as people may be more likely to use facilities in the wider city landscape. We also presented data showing that the availability to four key facilities within a fixed neighbourhood boundary may not be a useful proxy of visits for children living in urban environments. They may be more likely to use facilities elsewhere in the wider city scape. We suggested, like others (Chaix et al., 2017; Patterson and Farber, 2015), that further methodological development is required to describe how features of the urban environment and individual's time/space geographies intersect. Therefore, we created a new landscape model to do this for 100 children living in the Central Belt of Scotland. The model we presented simultaneously assessed all the types of environments children could and did visit, without being constraint to fixed areal units.

This study also contributes to the literature around childhood mobility and environmental exposure. We found that the presence of specific land-uses across the urban landscape to be associated with the time spent by children there, for example libraries (IRR:7.40, 95% CI 2.13 to 25.68) and places of worship (IRR:5.98, 95% CI 3.33 to 10.72). We also observed a relationship between increased walkability of a cell and a greater time spent there. Children spent more time in cells containing playing fields, public parks and play parks, independently of proximity to their home. For most land-uses there were few sex differences in time spent there, although girls spent more at leisure centres time than boys (IRR:52.86, 95% CI 2.64 to 1058.56). Children living in more deprived areas spent more time at playing fields (IRR:1274.11, 95% CI 14.59 to 111301.72) and play parks (IRR:62.80, 95% CI 14.44 to 273.14) than children from less deprived areas. We have previously found more play parks in deprived areas in Glasgow compared to more affluent areas (Ellaway et al., 2007).

### 4.1 Environmental exposure and urban mobility

Other studies have combined GPS and land use data for a variety of different purposes, such as exploring activity spaces (Loebach and Gilliland, 2016), travel behaviour to/from home to school (Dessing et al., 2014), infectious disease probability mapping (Vazquez-Prokopec et al., 2013), potential physical activity opportunities (Wheeler et al., 2010), and exposure to fast food outlets (Sadler et al., 2016). Each study has contributed to the technological evolution of the field, but each largely concentrated on single aspects of the built environment and did not explore total land-use availability and use across a much wider geographical area. Our methodology adds an ability to utilise GPS data to explore the complexity of children's environmental exposures in a way that is not constrained to fixed neighbourhood boundaries around the home. The ability to quantify associations between different features of the built-environment and time spent there, allows an assessment of how urban design might affect where people go and what they do. The model we developed can be applied in future studies. Users should carefully consider the land-uses included within the model and grid cell size, if based upon the SDM principles. Further development of the models could consider the relationships between specific places of interest, for example key destinations within an urban area where individuals will spend time, and the structure of the urban landscape as a whole. For example, if parks are key areas of interest, what should their optimum spatial arrangement be, in order to maximise and equalise access? The underlying concept of encapsulating the entire urban landscape could also be used for describing objective exposure to health benefiting or damaging environments, such as unhealthy commodity retailing. We recommend future environmental exposure in health research searches a broad range of disciplines for environmental exposure models outside of those commonly used by epidemiologists.

### 4.2 Putting our landscape model into practice: what does it mean for children's health?

Whilst it is illuminating to see which environments children visit and linger in, it is also useful for health policy and for designing interventions. Roads and other transport network hubs, such as rail and bus stops, are associated with urban mobility and it is no surprise that our cohort of children living within the Scottish Central Belt, a largely urban area, were more likely to spend time within areas that contained these land uses, and within more walkable areas. These environments have been described as being both health benefiting, in terms of enabling mobility (Rydin et al., 2012), and health damaging. Health damaging and hazardous factors for children include greater risk from road traffic accidents as most child pedestrian injuries occur primarily in residential areas (Stevenson et al., 2015). Children from poorer households spend more time walking or playing near roads (Vaganay et al., 2003) and these children are five times as likely to die from road traffic accidents than children from wealthy backgrounds (Mackett and Thoreau, 2015).

Children may spend a greater amount of time at food and retail facilities as families increasingly tend to consume more food-away-from-home (Lee et al., 2016). The design of urban spaces in Western European countries for ‘family leisure’ (Karsten et al., 2015), has been transformed; the boundaries between eating, drinking, playing, informing and socialising have been purposefully eroded (Karsten et al., 2015). Although negative health impacts of increased food-away-from-home have been described, for example that childhood obesity rates in Western countries have increased dramatically (Lee et al., 2016), many retailers have responded to increased public demand by attempting to develop healthy eating environments for children (Lee et al., 2016). These kind of environments did attract children in our study and it therefore suggests greater effort is required by local and national governments to ensure that family leisure places are healthy environments (Wright et al., 2015).

Children also spent time at institutional facilities (including places of worship, libraries and recreational) and schools (Rasmussen, 2004). These settings can and are influenced by national policy that can support and encourage healthy behaviour changes (Jaime and Lock, 2009; Thornley et al., 2017). National and local policy makers should also consider the regulation of the content of advertising at public transport stops, a location children are more likely to spend time. A study of a large European city showed that advertising at 85% of transport stop locations was food, of this 40% for fast food and no adverts for fresh fruit or vegetables (Robertson et al., 2017).

Before adjustment for the home location and distance to home, our models found that private gardens were more attractive for children than many other land-uses. It was no surprise that this effect was lost once the home location was included within the model, suggesting that the private garden they spent most time in was theirs or that of a close neighbour. Studies have shown that having access to a private garden is negatively associated with sedentary time (Pulsford et al., 2013) and therefore healthy urban design policy should ensure that private outdoor space is incorporated into residential dwellings. The association with children spending time in grid cells containing playing fields, public parks and play parks *increased* once the models adjusted for home and school location, indicating that children are more likely to spend time there if they are not in the immediate home or school location. Research in Denmark showed that although access to and use of green space was high, distance to green space was not a limiting factor in use (Schipperijn et al., 2010). Children may visit local facilities based upon other factors, such as quality or facilities (Van Dillen et al., 2012), rather than being their closest park. Although we were unable to assess facility quality in our study, we recommend that future studies should.

### 4.3 Strengths and weaknesses

In this large study of 100 children residing across the central belt of Scotland, the children wore GPS monitors which recorded their precise locations at regular intervals allowing us to examine in detail their environmental exposure. A key strength of our study was the ability to include, and therefore adjust for, the environments that children did and could visit within the same model. Our study is novel in creating a detailed and fine scale model of urban landscape which described the presence of a range of land-uses that children could visit. We were able to explore the relationship between individual characteristics of children, such as sex and socio-economic status, and differences in the likelihood of whether they would spend more time at various land-uses. The children in our study were a sub-sample from a representative Scottish cohort, however those living within our study area and who wore GPS devices were a more affluent group; approximately 50% living in the most affluent areas of Scotland. This may limit the generalisability of the results but we did adjust the analysis for socio-economic position and our results will be generalisable for children living in the rest of the United Kingdom and for many Western countries. The sample of 100 children, although large for a GPS study, does limited the findings, particularly the interaction analyses exploring differences in the places children spent time by gender and socio-economic position where large confidence intervals were reported.

The number of days of GPS data collection is important and it has been suggested that up to 14 valid days of GPS monitoring may be needed to provide an accurate snapshot of ‘routine’ and reflect a range of environmental attributes individuals encounter (Zenk et al., 2018). We collected 7 days of GPS data which may be a limitation of our study, however a strength is that our data collection covered a full twelve-month period, therefore measuring mobility during different seasons.

As our underlying urban landscape model encompassed multiple local authorities, we relied on national datasets to obtain reliable and comparable facility and amenity location information, however these did not provide fine grained categories for all retailers. For example, ‘food and/or drink retail’ was a broad category and a more specific definition would have been more useful for understanding specific exposures.

We knew where children went and for how long, but not *why* they went there. For some land uses it was possible to guess at an activity, but there were few locations where we could be certain. Also, whilst we could show that children did not use their closest facility, we did not know why. Further qualitative research is required to understand the reasons for visiting land-uses. We included all GPS points, regardless of travel mode or length of time spent at a place. Although this will include instances where children were driven past a place, we chose to include all of the GPS points regardless of time spent to understand how the entire urban landscape is utilised. As the GPS receivers recorded at 10 s intervals, the total time spent within a grid cell when being driven will be small and this will be reflected in the statistical models. If children are driven past particular places often or spend a lot of time on roads, this is a significant exposure and place in which children spend time.

Our analyses did not explicitly allow for spatial autocorrelation in the data. We did include a crude spatial measure; distance of each grid cell to the child's home location (for all 100 children). To properly allow for the spatial structure of the data would have required a ‘trip’ or spatial-sequence approach to the models, which understood the chances of being in a cell at time *t* would be a function of location at time *t-1*. Indeed, the information to be gained from understanding the sequences of visits to different land covers/uses is likely to be substantial and will better reflect the complexity of both the urban landscape and urban life; this is where our work is now headed. Future research should develop methods to include a spatial structure across significantly large urban landscapes and explore the relationship between multi-purpose facilities (i.e. multi-use areas containing play parks, food and retail facilities), proximity to other facilities (i.e. the ability to drive to a nearby shopping complex after visiting a park), and quality of the environment. Our next step will be to explore how active children are in a particular setting.

### 4.4 Conclusions

Following calls from many academics, we developed and applied a new method to describe environmental exposure and urban mobility. This novel study used detailed mobility data of 10/11 year old children living in Scotland to explore whether they spent more or less time at various land-uses. In addition to the presentation of methods, the results have important policy implications and highlight land-uses across a wide urban area, that regardless of distance from home, children will spend more or less time at. We found that females spend more time at leisure centres than boys and children from deprived households will spend more time at playing fields and play parks than children living in more affluent areas. Our findings can support health public policy by highlighting spaces children spend more time. Our findings clearly align with public health policies, such as for obesity promotion, that for any sustainable and beneficial effect of public health interventions time spent at home and in school must be included as a key factor (Lobstein et al., 2015).

The ability to consider how children use their urban area, and the multiple environments they are exposed to, is a significant step towards understanding the urban environment as a complex system.

## Conflict of interest statement

The authors declare that there are no conflicts of interest.

## Data sharing statement

For further information, please refer to the SPACES study data sharing portal at http://spaces.sphsu.mrc.ac.uk/.

## Funding statement

JO, RM, PM and AE are employed by the University of Glasgow and funded as part of the Neighbourhoods and Communities Programme (MC_UU_12017/10) (SPHSU10) at the MRC/CSO Social and Public Health Sciences Unit (SPHSU).
