# Supplementary material for: Children's mobility and environmental exposures in urban landscapes: A cross-sectional study of 10–11 year old Scottish children
Source: Soc Sci Med. 2019 Mar;224:11–22. doi: 10.1016/j.socscimed.2019.01.047 (PMC6411928; doi:10.1016/j.socscimed.2019.01.047)
Supplement: Multimedia component 1 [file mmc1.docx]

| **Variable** | **Unadjusted** | | | | **Adjusted for home & school** | | | | **Adjusted for home, school and distance from home** | | | |
| --- | --- | --- | --- | --- | --- | --- | --- | --- | --- | --- | --- | --- |
|  | **IRR** | **P** | **LL 95% CI** | **UL 95% CI** | **IRR** | **P** | **LL 95% CI** | **UL 95% CI** | **IRR** | **P** | **LL 95% CI** | **UL 95% CI** |
|  | | | | | | | | | | | | |
| Dense population | 1.10 | 0.69 | 0.69 | 1.76 | 0.97 | 0.88 | 0.67 | 1.41 | 1.21 | 0.42 | 0.76 | 1.93 |
|  | | | | | | | | | | | | |
| Urban (6 fold 1&2) | 2.69 | 0.01 | 1.29 | 5.59 | 4.65 | 0.00 | 2.78 | 7.80 | 2.95 | 0.00 | 1.92 | 4.52 |
|  | | | | | | | | | | | | |
| Income SIMD | | | | | | | | | | | | |
| 1 (Most deprived) | REF | | | | REF | | | | REF | | | |
| 2 | 2.15 | 0.06 | 0.96 | 4.80 | 1.33 | 0.25 | 0.82 | 2.13 | 1.20 | 0.50 | 0.70 | 2.04 |
| 3 | 4.17 | 0.00 | 1.85 | 9.38 | 2.87 | 0.00 | 1.73 | 4.78 | 2.62 | 0.00 | 1.46 | 4.70 |
| 4 | 6.71 | 0.00 | 3.66 | 12.31 | 4.83 | 0.00 | 2.66 | 8.75 | 2.32 | 0.00 | 1.31 | 4.10 |
| 5 (Least deprived) | 3.97 | 0.00 | 2.07 | 7.62 | 4.72 | 0.00 | 2.69 | 8.30 | 3.13 | 0.00 | 1.68 | 5.85 |
|  | | | | | | | | | | | | |
| B or minor road | 2.74 | 0.00 | 2.01 | 3.72 | 2.71 | 0.00 | 2.05 | 3.58 | 1.82 | 0.00 | 1.33 | 2.51 |
|  | | | | | | | | | | | | |
| Motorway or A road | 2.86 | 0.00 | 1.95 | 4.19 | 7.07 | 0.00 | 5.24 | 9.53 | 26.42 | 0.00 | 7.34 | 95.03 |
|  | | | | | | | | | | | | |
| Railway stop | 2.87 | 0.01 | 1.24 | 6.66 | 5.79 | 0.00 | 2.58 | 12.97 | 3.89 | 0.00 | 1.59 | 9.49 |
|  | | | | | | | | | | | | |
| Bus stop | 1.27 | 0.22 | 0.87 | 1.85 | 3.52 | 0.00 | 2.32 | 5.34 | 1.92 | 0.00 | 1.46 | 2.53 |
|  | | | | | | | | | | | | |
| Walkability score | | | | | | | | | | | | |
| 1 (Least walkable) | REF | | | | REF | | | | REF | | | |
| 2 | 7.27 | 0.00 | 4.31 | 12.24 | 5.97 | 0.00 | 3.81 | 9.38 | 4.85 | 0.00 | 3.23 | 7.28 |
| 3 | 13.99 | 0.00 | 8.81 | 22.21 | 9.64 | 0.00 | 6.15 | 15.10 | 9.83 | 0.00 | 4.88 | 19.79 |
| 4 | 14.40 | 0.00 | 7.07 | 29.35 | 8.81 | 0.00 | 5.31 | 14.60 | 5.89 | 0.00 | 3.40 | 10.20 |
| 5 (Most walkable) | 17.74 | 0.00 | 8.59 | 36.64 | 18.72 | 0.00 | 10.65 | 32.89 | 10.50 | 0.00 | 5.93 | 18.57 |
|  | | | | | | | | | | | | |
| Food and/or drink retail | 1.59 | 0.15 | 0.85 | 2.98 | 4.90 | 0.00 | 3.08 | 7.79 | 4.12 | 0.00 | 2.36 | 7.20 |
|  | | | | | | | | | | | | |
| Leisure Centre | 4.55 | 0.00 | 2.41 | 8.59 | 8.43 | 0.00 | 4.81 | 14.76 | 13.93 | 0.14 | 0.44 | 443.52 |
|  | | | | | | | | | | | | |
| Place of worship | 4.41 | 0.00 | 2.69 | 7.23 | 13.10 | 0.00 | 6.46 | 26.56 | 6.32 | 0.00 | 3.36 | 11.88 |
|  | | | | | | | | | | | | |
| Library | 26.88 | 0.00 | 11.93 | 60.54 | 37.55 | 0.00 | 13.36 | 105.53 | 7.40 | 0.00 | 2.63 | 20.78 |
|  | | | | | | | | | | | | |
| Derelict land | 0.24 | 0.00 | 0.10 | 0.60 | 0.38 | 0.00 | 0.21 | 0.70 | 0.44 | 0.01 | 0.24 | 0.81 |
|  | | | | | | | | | | | | |
| Private Gardens | 4.27 | 0.00 | 2.98 | 6.11 | 1.27 | 0.09 | 0.96 | 1.68 | 0.80 | 0.13 | 0.61 | 1.06 |
| Playing field | 2.96 | 0.00 | 1.89 | 4.65 | 4.70 | 0.00 | 2.98 | 7.39 | 244.39 | 0.10 | 0.37 | 159289.73 |
| Sports club | 0.81 | 0.45 | 0.46 | 1.41 | 2.08 | 0.06 | 0.98 | 4.41 | 1.36 | 0.32 | 0.74 | 2.53 |
| Woodland | 0.27 | 0.00 | 0.19 | 0.38 | 0.44 | 0.00 | 0.32 | 0.60 | 0.40 | 0.00 | 0.30 | 0.52 |
| Public park | 2.63 | 0.00 | 1.66 | 4.17 | 5.27 | 0.00 | 3.11 | 8.93 | 6.06 | 0.07 | 0.90 | 40.98 |
| Play park | 1.11 | 0.81 | 0.49 | 2.51 | 3.93 | 0.02 | 1.24 | 12.48 | 1.75 | 0.31 | 0.59 | 5.22 |
| Green verge | 0.49 | 0.05 | 0.24 | 1.01 | 0.56 | 0.03 | 0.34 | 0.93 | 0.72 | 0.24 | 0.42 | 1.24 |
| Other | 0.97 | 0.92 | 0.61 | 1.57 | 1.44 | 0.05 | 1.00 | 2.09 | 1.18 | 0.34 | 0.84 | 1.67 |
|  |  |  |  |  |  |  |  |  |  |  |  |  |
| School (polygon) |  |  |  |  | 94965.60 | 0.00 | 42899.27 | 210222.13 | 10477.93 | 0.00 | 4860.79 | 22586.33 |
|  | | | | | | | | | | | | |
| Home (50m of postcode) |  |  |  |  | 243441.03 | 0.00 | 162614.88 | 364441.02 | 42552.76 | 0.00 | 25008.47 | 72404.95 |
|  | | | | | | | | | | | | |
| Distance from home (m) |  |  |  |  |  |  |  |  | 0.99983 | 0.00000 | 0.99977 | 0.99989 |

**Supplementary Table 1: Land-uses within grid cells associated with children spending time there (unadjusted).**
